# Hard Times

**Published:** 1858

**Authors:** 


HARD TIMES.—Look at our receipt list and you will be satisfied that we have not erred in our diagnosis. We will [illegible] venture a prognosis until our next issue.

